# Microbiological and Clinicoepidemiological Profile of a Series of Patients with Infective Endocarditis at a Center in Eastern Nepal

**DOI:** 10.1155/2021/9980465

**Published:** 2021-07-22

**Authors:** Sohani Bajracharya, Basudha Khanal, Shraddha Siwakoti, Rupa Rajbhandari Singh, Sanjib Kumar Sharma

**Affiliations:** ^1^Department of Microbiology & Infectious Diseases, B. P. Koirala Institute of Health Sciences (BPKIHS), Dharan 56700, Sunsari, Nepal; ^2^Department of Pediatrics, B. P. Koirala Institute of Health Sciences (BPKIHS), Dharan 56700, Sunsari, Nepal; ^3^Department of Internal Medicine, B. P. Koirala Institute of Health Sciences (BPKIHS), Dharan 56700, Sunsari, Nepal

## Abstract

**Background:**

The microbiological and clinicoepidemiological profile of infective endocarditis (IE) has undergone significant change over time. The pattern of IE studied at local level provides broader vision in understanding the current scenario of this disease. This study aimed to depict the overall picture of IE and its changing profile by evaluating the microbiological and clinicoepidemiological features in the context of a tertiary care center of eastern Nepal.

**Methods:**

The descriptive study was conducted from September 2017 to August 2018 among IE patients presenting to B. P. Koirala Institute of Health Sciences, Nepal. Detailed history and clinical manifestations of patients were noted. Microorganisms isolated from the blood culture were processed for identification by standard microbiological methods, and susceptibility testings were done. Each patient was assessed daily during hospital stay.

**Results:**

Ten definite and 7 possible endocarditis cases were studied. The mean age was 41.4 ± 15.85 (17–70) years with predominance of male (4.7 : 1). Rheumatic heart disease (41.1%) was the most common underlying heart disease observed followed by injection drug user endocarditis (23.5%). All the cases had native valve endocarditis. Aortic valve was the most common valve involved (35.3%) followed by mitral, tricuspid, and pulmonary valves. Blood culture positivity was 53%. *Staphylococcus aureus* was the major causative agent responsible for 23.5% of the cases followed by *Enterococcus faecium*, *Enterococcus faecalis*, and *Pseudomonas aeruginosa*. Mortality of 2 cases (11.8%) was associated with *S. aureus* and *P. aeruginosa*. Majority of patients developed acute kidney injury (35.3%) and congestive cardiac failure (23.5%).

**Conclusion:**

IE patients in our center exhibited differences from the west in terms of age at presentation and predisposing factors but held similarity in terms of commonly isolated microorganisms. The changing patterns of IE, etiological agents, and their antimicrobial susceptibility observed in this study may be helpful for clinicians in formulating a new empirical antibiotic treatment protocol.

## 1. Introduction

Infective endocarditis (IE) is a life-threatening condition that involves infection of endocardial surface of the heart. It is a relatively uncommon disease but still poses a major health risk due to its increasing morbidity and mortality [1].

The clinical, epidemiological, and microbiological profiles of IE vary globally [2, 3]. In recent decades, several studies have noted an increase in the proportion of IE caused by *Staphylococcus aureus* [1, 4–6]. *Staphylococcus* scoring over *Streptococcus* group could be due to changing predisposing factors such as intravenous (IV) drug abuse, prosthetic valves, surgical interventions, and healthcare-associated infections [4, 7, 8]. However, rheumatic heart disease (RHD) still continues to remain as an important predisposing factor in developing nations [5, 6, 9, 10]. IE in the urban world has often been seen in IV drug users, hemodialysis patients, and patients with prosthetic valves [11]. IE is associated with prolonged hospitalization: some require surgeries and others may develop cardiovascular, neurological, or renal complications [12, 13]. Proper understanding of IE epidemiology is hence important to determine the impact of disease and amend treatment plans.

Abundant studies have highlighted various aspects of IE in developed nations; however; limited data are available on this condition in the developing countries particularly in Nepal [5, 14]. In view of changing the face of IE, there was a need to collect data from local level to recognize the nature of disease and update the existing empirical treatment policy. Hence, this study was conducted to evaluate the microbiological and clinicoepidemiological profile of IE in a tertiary care center in eastern Nepal.

## 2. Materials and Methods

### 2.1. Study Design and Population

A hospital-based descriptive prospective study was carried out in the Department of Microbiology, B. P. Koirala Institute of Health Science (BPKIHS), Dharan, Nepal, for a period of a year from September 2017 to August 2018. The study was conducted in collaboration with the Department of Internal Medicine and Department of Pediatrics.

### 2.2. Patients' Screening

All the admitted patients with clinical suspicion of IE were included in the study. Detailed history and clinical manifestations from the consenting patients were taken and recorded in the predefined proforma. Each patient was observed and assessed daily for the progression of disease and improvement. Clinical details of the patient and any complications seen were noted. Blood samples for culture and sensitivity were taken from all the patients. All of them had undergone echocardiogram.

Patients were categorized as definite IE and possible IE on the basis of Duke's major and minor criteria [15]. Those patients who did not fulfill Duke's criteria for definite and possible IE were excluded from the study. On the basis of Duke's criteria [15], IE is categorized as definite IE with clinical features meeting two major criteria or one major and three minor criteria or five minor criteria. Possible IE are cases fulfilling one major and one minor criteria or three minor criteria. Rejected IE are those with alternate diagnosis established or resolution of the infection with antibiotic treatment for <4 days or no pathologic evidence of IE at surgery or autopsy after 4 days of antibiotic therapy or less.

The major criteria are (i) two separate positive blood cultures of typical microorganisms of IE, such as *Viridans streptococci, Streptococcus bovis*, HACEK group, *Staphylococcus aureus* or community-acquired *Enterococci* in the absence of a primary focus, or persistently positive blood cultures drawn >12 hrs apart or all of three or a majority of four or more separate blood cultures, with first and last drawn at least 1 hr apart and (ii) evidence of endocardial involvement as shown by echocardiogram with an oscillating intracardiac mass on valve or supporting structures or in the path of regurgitant jets or in implanted material, in the absence of an alternative anatomic explanation or abscess or new partial dehiscence of prosthetic valve or new valvular regurgitation.

The minor criteria are (i) predisposing heart conditions or injection drug use; (ii) fever ≥38.0°C (≥100.4°F); (iii) vascular phenomena: major arterial emboli, septic pulmonary infarcts, mycotic aneurysm, intracranial hemorrhage, conjunctival hemorrhages, and Janeway lesions; (iv) immunologic phenomena: glomerulonephritis, Osler's nodes, Roth's spots, and rheumatoid factor; and (v) microbiologic evidence: positive blood culture but not meeting major criterion as noted previously or serologic evidence of active infection with organism consistent with infective endocarditis.

### 2.3. Blood Culture Collection and Processing

Blood cultures were drawn at the time of admission before the start of antibiotics whenever possible by applying standard precautions. Three separate venous blood samples were obtained from 3 different venipuncture sites. The time interval between two successive samples collection was at least 30 minutes to 1 hour apart to allow proof that the bacteremia was continuous [16]. Blood samples collected were placed either in brain heart infusion broth (BHIB) or BD BACTEC culture vials. The use of conventional or automated system depended on clinicians' decision. Blood samples obtained in BHIB were incubated for 3 weeks with periodic gram stains and subcultures [17]. Blood samples obtained in BACTEC Plus Aerobic vials were placed into BACTEC 9050 blood culture system for 5 days. The identification of pathogens was done by standard microbiological techniques using the conventional method of characterization [18]. Antimicrobial susceptibility testing was performed on Mueller–Hinton agar (HiMedia Laboratories) by the Kirby–Bauer's disc diffusion method as recommended by Clinical and Laboratory Standards Institute (CLSI) guidelines. Staphylococcal strains were screened for methicillin resistance using the oxacillin agar plate method [19]. Susceptibility to vancomycin for *S. aureus* and susceptibility to colistin for *P. aeruginosa* were tested by determining minimum inhibitory concentration (MIC) by the agar dilution technique [20].

### 2.4. Statistical Analysis

Data were collected and entered into a database using MS Excel 2013. Statistical analysis was conducted using SPSS version 13. The chi-square test was applied for comparison of categorical variables. The *T*-test was applied for comparison of mean values of two independent samples. *P* value less than 0.05 was considered as statistically significant.

## 3. Results

A total of 24 patients were studied. Ten cases (41.7%) were classified as definite endocarditis and 7 cases (29.2%) as possible endocarditis based on Duke's major and minor criteria. The remaining 7 cases (29.2%) did not fulfill Duke's criteria and were excluded from the study. Of those 10 cases with definite IE, 5 satisfied both major Duke's criteria and remaining 5 had one major and three minor criteria. All of 7 patients with possible endocarditis had one major and two minor criteria.

In this study, a total of 17 patients in the age range of 17–70 years with the predominance of male (4.7 : 1) were seen. The mean age of patients was 41.4 ± 15.85 years.

Eleven patients (64.7%) were below 45 years of age, and 3 (17.6%) were elderly patients (>65 years) with no pediatrics cases.

Clinical features, possible predisposing conditions, and site of vegetation are depicted in Table 1.

No previous comorbidities such as hypertension, diabetes mellitus, degenerative valve disease, and hemodialysis were seen on any of the patients. Congenital heart disease and history of drug abuse are shown in Table 1. History of dental procedure was not present in any of the cases.

All of the cases had native valve endocarditis with different sites of vegetation as mentioned in Table 2. Vegetation implanted on tricuspid valve was all associated with intravenous drug abuse-related IE. Majority of the rest of left-sided endocarditis were due to RHD and few due to other cardiac conditions.

Blood culture positivity was 53%. There was one case of mixed infection. *Staphylococcus aureus* was the major causative agent with 2 methicillin- sensitive *Staphylococcus aureus* (MSSA) and 3 methicillin-resistant *Staphylococcus aureus* (MRSA) strains. *Enterococcus* spp. and *Pseudomonas aeruginosa* were the other isolates (Table 3).

Among 4 cases with history of intravenous drug abuse, 3 cases were culture positive. Two cases had *S. aureus* as the causative agent and *E. faecalis* in the remaining one. Among 4 of enterococcal IE cases, one of the female patients had history of urinary tract infection and another 68-year-old male patient had history of transurethral resection of the prostate. Similarly, *Pseudomonas aeruginosa* was isolated from a critically ill patient who had chronic kidney and liver disease and was undergoing dialysis routinely.

Treatments of all 17 cases were as per clinicians' advice and their antimicrobial susceptibility pattern. *Staphylococcus aureus* was variably treated with gentamicin, teicoplanin, cloxacillin, and vancomycin. *Enterococcus* spp. were treated with teicoplanin and ampicillin + sulbactam. A single isolate of *Pseudomonas aeruginosa* was multidrug resistant and was treated initially with reserved antimicrobials such as piperacillin + tazobactam and imipenem, which were later changed to colistin. Antibiotic susceptibility pattern of the IE isolates is depicted in Table 4.

A single isolate of *Pseudomonas aeruginosa* was susceptible only to colistin and was resistant to piperacillin, piperacillin + tazobactam, ceftazidime, cefepime, aztreonam, imipenem, tobramycin, ciprofloxacin, amikacin, ofloxacin, and levofloxacin.

**Figure 1:**
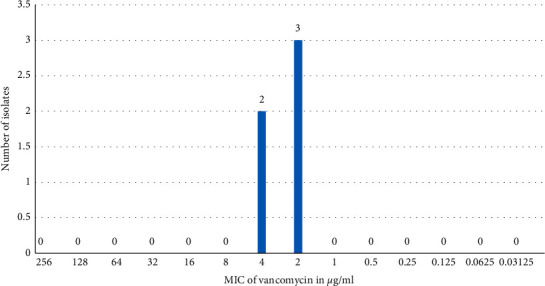
MIC of vancomycin for *Staphylococcus aureus* (*n* = 5).

MIC of vancomycin for *Staphylococcus aureus* revealed 3 vancomycin-sensitive *Staphylococcus aureus* (VSSA) and 2 vancomycin intermediate *Staphylococcus aureus* (VISA) strains (Figure 1).

Complications during hospital stay were seen in 12 patients (70.6%), as shown in Table 5. Majority of patients developed acute kidney injury (AKI). AKI in half of the patients was attributed to the drug used during the treatment aminoglycoside, to be particular gentamicin. Among the etiological agents of IE in our study, *S aureus* was associated with complications in maximum of the cases (Table 6). The length of hospital stay ranged from 2 to 60 days. This variability in the duration of hospital stay was due to the course of events during hospital stay, patients leaving against medical advice and patient being referred to other centers either due to lack of ICU bed or for surgical intervention of vegetation. However, all the patients were evaluated till the time of discharge. There were 2 cases (11.8%) of in-hospital mortality associated with *S. aureus* and *P. aeruginosa*.

History of prior antibiotic intake was seen in 6 (35.3%) amongst 9 culture positive cases and 7 (41.2%) amongst 8 culture negative cases with total of 13 cases (76.5%). While comparing culture-positive and culture-negative cases, prior antibiotic intake was not found to be statistically significant factor (*P*=0.312) (Table 7).

## 4. Discussion

A total of 17 patients with suspicion of IE were enrolled in our study. Ten patients (41.7%) had definite IE and 7 (29.2%) had possible IE. Diagnosis of very few cases as IE in our setting indicates its low incidence as shown in some other studies [5, 6, 10]. Absence of pediatric case in this study is in accordance to fewer rates of IE in pediatric age group as reported elsewhere [21, 22]. The low incidence of IE in this study might have failed to comprise pediatric IE cases which is itself rare in children. This study showed male predominance with the ratio of male to female being 4.7 : 1, which is comparable to studies in India and Pakistan [4, 6, 10, 23]. The mean age of patient was 41.4 ± 15.85 years, similar to results of studies done in Oman (43.6 yr) [24], Iran (45 yr) [25], and China (47.8 yr) [26]. However, this mean age was older than the mean age in studies previously done by Ghimire et al. (27.3 yr) [5] and Sherpa et al. (31 yr) [14] from this institution. This increase in mean age of patients over time indicates late onset of disease and change in trend of IE presentation from early 30 s to early 40 s. However, this is contrary to studies in the west and developed nations where the incidence of IE is higher in 5^th^ to 6^th^ decade of life [6, 27]. The proportion of elderly patients (>65 years) in this study was 17.6%, while it was reported to be about 50% in western studies [1, 27]. In this regard, it suggests that IE is still common in younger population in Nepal.

RHD accounted for the most common predisposing factor, which was consistent with the findings of several studies [3, 4, 6, 10, 23]. RHD usually presents in an early age; hence, the younger individuals are more prone to IE. Half of the age groups with RHD were below 45 yrs of age in this study. Also limited facilities for surgical correction may have contributed to continued occurrence of RHD in our part of world. IVDU as a risk factor for IE was seen in 4 patients (23.5%) in this study. Studies conducted in Southeast Asia showed variable number of patients with history of IVDU such as 9% by Ghosh et al. [6], 1.6% by Gupta et al. [10], and 8% by Khan et al. [4]. However, some studies showed no such risk factor [9, 23]. This variability could be due to more number of IVDUs or less number of total cases.

This study had all the cases with native valve endocarditis. General preference of Nepalese to medical rather than surgical intervention due to low socioeconomic status could be one fact that prosthetic valve endocarditis is less in number. Also, follow-up of postoperative prosthetic valve patients at a center with advanced medical care could be another reason [6]. Higher proportion of patients with aortic valve involvement as seen in this study was similar to the studies by Netzer et al. [28] and Loupa et al. [29]. Many studies have reported mitral as the frequently involved valve followed by aortic and tricuspid [3, 4, 6, 25]. However, both mitral and aortic valves are vulnerable to the deforming effect of rheumatic activity.

Regarding clinical features, fever was the most common symptom similar to previous study by Sherpa et al. [14]. Classical peripheral manifestations of IE such as petechiae, splinter hemorrhage, and septic emboli were seen. Osler's nodes, Janeway lesions, and Roth's spots were not observed as these are relatively rare [30].

Blood culture was positive in 9 patients (53%). This percentage is in accordance to findings of Tariq et al. [31] and Khan et al. [4]. Contrary to that, more than 80% positive blood cultures have been reported in the western series [27, 32–34]. Endocarditis with negative blood culture occurs mostly due to the initiation of antibiotic therapy before culture and infection with highly fastidious bacteria or nonbacterial pathogens [25]. Cases of blood culture negative infective endocarditis (BCNIE) ranged from 30 to 50% in studies conducted in India and Pakistan [4, 10]. In this study, the frequency of BCNIE was 47%, similar to our neighboring countries. In a tertiary care center like us, most patients encountered are referred cases who have probably received treatment elsewhere. Hence, history of prior antibiotic intake is high. However, when culture-positive cases were compared with culture-negative cases in this study, prior antibiotic therapy was not found to be a statistically significant factor (*P*=0.312). This finding is in accordance with the study by Gupta et al. [10] but in contrast to the study by Tariq et al. [35], Choudhury et al. [36], Garg et al. [37], and Khan et al. [4], where significant association was found between prior antibiotic therapy and incidence of culture-negative endocarditis.

*Staphylococcus aureus* as the predominant organism for IE as seen in this study has been reported by several recent studies [4–6, 10, 14], which differs from the previous common trend of *Streptococci* isolation. This suggests a change in the spectrum of causative agents of IE in our region. Due to the increase in exposure to invasive procedures related to urinary tract and gastrointestinal systems, *Enterococcal* IE is on the rise [27]. Among the *Enterococcal* IE cases seen in this study, a female patient had urinary tract infection and one of the 68-year-old male patient had history of transurethral resection of the prostate (TURP). *Pseudomonas aeruginosa* is a rare cause of IE and difficult to diagnose, and it results to high mortality [38]. There was also a single case of *Pseudomonal* IE in this study who succumbed to death.

All the complications described in our study have been previously reported in other studies too [14, 24, 35]. Congestive cardiac failure was the most common complication seen in many studies [1, 10, 26, 35]. However, the finding of our study matches with Sherpa et al. [14], a study previously done in the same hospital indicating that drug-induced AKI continue to occur in patients undergoing treatment of IE in eastern Nepal. IE patients with neurological, cardiac, and renal complications or sepsis or nosocomial endocarditis have been associated with an increased mortality as shown in the study by Tariq et al. [35]. This statement holds true to our study too as the causative agent for one of the two mortalities observed was *Pseudomonas*, which was seen as nosocomial infection. Similarly, mortality of the other case was also associated with another important nosocomial agent MRSA. *Staphylococcal* IE was found to have worse prognosis in a study by Hill et al. where it attributed to mortality of 33% [27]. Other studies have also shown *S. aureus* as an independent predictor of mortality [39–41]. But only a single mortality due to *S. aureus* was observed in our study. However, all of *Staphylococcal* IE cases developed complications during hospital stay indicating its association with poor clinical course.

As for the limitation of the study, the incidence of this disease is itself low so we faced the problem of limited sample size. Also, this study was conducted only for a year, had it been studied for a longer duration; limitation on sample size would not have been a problem. This study relied on the conventional method for identifying organisms which might have resulted in failure in the detection of organisms that could have been detected through serological or molecular techniques. Another limitation of the study is in regard to the outcome of patients as they were followed up only until the time of discharge. No follow-up of patients was made for those being referred to other centers. Thus, our study might have missed the true outcome of the patients.

## 5. Conclusion

This study has revealed the overall scenario of IE in the context of a tertiary care center in Nepal. Younger age at presentation and RHD as the common predisposing factor still remain the same in our region. However, increasing mean age, change in microbiological agent, and increasing IVDU-related IE depict the changing pattern of IE as seen in the west. Blood cultures grown were positive in half of the cases, with *Staphylococcus aureus* as the major isolate. The frequency of culture negative endocarditis (CNE) continues to remain high (47%), largely due to history of prior antibiotic intake (76.4%) in our series of 17 IE patients. The incidence of major complications related to *S. aureus* IE was 23.5% and 29.4% in CNE. We conclude that although it is a small study, disparities in different parameters hold value in understanding IE and diagnosing and formulating new empirical treatment protocol in our setup. However, multicenter hospital-based studies with large population in longer time frame are needed to draw out the complete picture of IE in Nepal.

## Figures and Tables

**Table 1:** Summary of clinical profiles of IE patients (*n* = 17).

Variables	Number (%)
*Gender*	
Male	14 (82.4)
Female	3 (17.6)

*Clinical features*	
Fever	16 (94.1)
Murmur	14 (82.4)
Malaise	12 (70.6)
Shortness of breath	11 (64.7)
Clubbing	10 (58.8)
Pallor	9 (52.9)
Arthralgia	8 (47)
Cough	7 (41.2)
History of weight loss	7 (41.2)
Splenomegaly	6 (35.3)
Chest pain	3 (17.6)
Petechiae	2 (11.8)
Seizure	1 (5.9)
Septic emboli	1 (5.9)
Splinter hemorrhage	1 (5.9)

*Age groups in years*	
0–20	1 (5.9)
20–40	7 (41.1)
40–60	6 (35.3)
>60	3 (17.6)

*Potential risk factors*	
Rheumatic heart disease	7 (41.1)
Intravenous drug abuse	4 (23.5)
Cardiomyopathy	2 (11.8)
Bicuspid aortic valve	2 (11.8)
Situs inversus dextrocardia	1 (5.9)
Risk factor unknown	1 (5.9)

**Table 2:** Size and site of vegetation detected by echocardiography in IE patients (*n* = 17).

Variables	Number (%)
*Size of vegetation*	
Size of ≥10 mm	9 (52.9)
Size of ≥20 mm	1 (5.9)
Mobile vegetation	5 (29.4)
No vegetation	2 (11.8)

*Site of vegetation*	
Aortic valve	6 (35.3)
Mitral valve	3 (17.6)
Tricuspid valve	3 (17.6)
Aortic + mitral valves	2 (11.8)
Pulmonary valve	1 (5.9)
No vegetation	2 (11.8)

**Table 3:** Microorganisms isolated from blood cultures in IE patients (*n* = 17).

Organism	Number of patients (%)
*Staphylococcus aureus*	4 (23.5)
*Enterococcus faecium*	2 (11.8)
*Enterococcus faecalis*	1 (5.9)
*Pseudomonas aeruginosa*	1 (5.9)
*Staphylococcus aureus and Enterococcus faecalis*	1 (5.9)
No growth on culture	8 (47)

**Table 4:** Blood culture and antimicrobial susceptibility pattern of 17 IE patients.

Organisms isolated	*S. aureus*	*E. faecalis*	*E. faecium*
No. of isolates	5	2	2

*Antibiotics tested and their susceptibility (S/R*, *S* *=* *susceptible*, *R* *=* *resistant)*			
Penicillin	0/5	2/0	0/2
Ampicillin		2/0	1/1
Amikacin	4/1		
Erythromycin	0/5	0/2	0/2
Ofloxacin	2/3		
Tetracycline	4/1	0/2	0/2
Cotrimoxazole	3/2		
Ceftriaxone	2/3		
Chloramphenicol	5/0		1/1
Linezolid	5/0	2/0	2/0
Amoxycillin + clavulanic acid	0/5		
Gentamicin	3/2		
Vancomycin		2/0	2/0
Teicoplanin		2/0	2/0
Ciprofloxacin		0/2	0/2
High-level gentamicin		1/1	1/1

**Table 5:** Complications and outcome of IE patients (*n* = 17).

Complications	Number (%)	Outcome of patients	Number (%)
Acute kidney injury (AKI)	3 (17.6)	Improved	10 (58.8)
Congestive cardiac failure (CCF)	1 (5.9)	Not improved/referred^*∗*^	3 (17.6)
Septic pulmonary emboli	1 (5.9)	Mortality	2 (11.8)
Cerebrovascular accident	3 (17.6)	Leaving against medical advice	2 (11.8)
AKI with CCF	3 (17.6)		
UGI bleeding	1 (5.9)		
No complications	5 (29.5)		

^*∗*^Referred to other medical centers due to lack of ICU beds and need of ventilatory support.

**Table 6:** Complications of patients with respect to the causative agents of IE (*n* = 17).

Causative agent (*n*)	Complications	
	Present, *n* (%)	Absent, *n* (%)
*S. aureus* (4)	4 (23.5)	0 (0.0)
*E. faecalis* (1)	0 (0.0)	1 (5.9)
*E. faecium* (2)	1 (5.9)	1 (5.9)
*P. aeruginosa* (1)	1 (5.9)	0 (0.0)
Mixed growth (1) *(S. aureus* + *E. faecalis)*	1 (5.9)	0 (0.0)
Culture negative (8)	5 (29.4)	3 (17.6)

**Table 7:** Comparison of culture-positive and culture-negative IE patients with history of prior antibiotic intake (*n* = 17).

IE patients	History of prior antibiotic intake		*P* value
	Present, *n* (%)	Absent, *n* (%)	
Culture positive (9)	6 (35.3)	3 (17.6)	0.312
Culture negative (8)	7 (41.2)	1 (5.9)	

## Data Availability

The data obtained from the study are available from the corresponding author upon request.

## Abbreviations

HACEK: *Haemophilus, Actinobacillus, Cardiobacterium, Eikenella, Kingella*
ICU: Intensive care unit
UGI: Upper gastrointestinal.
